# Efficacy of Manuka honey eye drops in managing dry eye disease after cataract surgery: a prospective controlled study

**DOI:** 10.3389/fopht.2026.1812914

**Published:** 2026-04-28

**Authors:** Javier García-Bardera, Javier García-Bella, Fiorella K. Cuba-Sulluchuco, Bárbara Burgos-Blasco, Pedro Arriola-Villalobos, Jose Manuel Benítez-Del-Castillo

**Affiliations:** Ophthalmology Department, Hospital Clinico San Carlos, Madrid, Spain

**Keywords:** catquest, conjunctival redness, K5M, NIBUT, optimel, OSDI

## Abstract

**Background:**

To evaluate the efficacy of Leptospermum spp (Manuka) honey eye drops in improving dry eye symptoms and reducing ocular inflammation after cataract surgery, compared to sodium hyaluronate eye drops.

**Methods:**

A prospective controlled study including 53 eyes undergoing cataract surgery was conducted. Patients were consecutively allocated to Manuka eye drops (n = 25) or sodium hyaluronate (n = 28). Subjective symptoms were assessed using the Ocular Surface Disease Index (OSDI) and CATQUEST-SF9 questionnaires. Objective parameters included non-invasive tear break-up time (NIBUT) and conjunctival redness measured with the Keratograph^®^ 5M. Assessments were performed preoperatively and at 1 day, 1 week, and 1 month.

**Results:**

A total of 53 eyes from 53 patients (18 males and 35 females) were analyzed, with a mean age of 72.1 ± 7.6 years. At 1 month, the Manuka group showed a greater reduction in OSDI score compared to the control group (-27.3 ± 20.3 vs -4.3 ± 17.4). After adjustment for baseline OSDI and sex, OSDI remained significantly lower in the Manuka group (adjusted mean difference: -18.7; 95% CI: -28.8 to -8.7; p = 0.007). Conjunctival redness was also lower in the Manuka group at 1 month (adjusted mean difference: -0.56; 95% CI: -0.91 to -0.21; p = 0.014). No significant differences were observed in NIBUT between groups.

**Conclusion:**

Manuka eye drops were associated with greater improvement in postoperative dry eye symptoms and reducing ocular surface inflammation compared to sodium hyaluronate. These findings should be interpreted with caution and considered hypothesis-generating.

## Introduction

Dry eye is a complex and multifactorial disease of the ocular surface characterized by symptoms such as discomfort and visual disturbances which collectively contribute to a decrease in patients’ quality of life. According to the 2017 Tear Film and Ocular Surface Society (TFOS) Dry Eye Workshop (DEWS) II, dry eye disease (DED) is one of the most common reasons for consultation with a general ophthalmologist, yet, despite its prevalence, there remains no definitive cure (1).

It is estimated that approximately 34% of the global population is affected by this condition, with prevalence rising to as high as 75% in certain groups and increasing with age (2). Hence, it is important to understand the contributing factors, which include inflammation, neurosensory abnormalities, environmental triggers, and iatrogenic causes. Among the last group, cataract surgery is a well-known intervention that can both induce and exacerbate dry eye symptoms (3). Symptom exacerbation typically reaches its peak within the first day postoperatively and may persist for a duration ranging from 1 to 12 months following the surgical intervention. It has been estimated that approximately 34-37% of patients undergoing cataract surgery continue to experience persistent symptoms resembling dry eye disease (DED) at 6 months post-surgery (4).

Currently, the management of dry eye disease focuses on alleviating symptoms and improving the patient’s overall ocular surface health through a combination of non-pharmacologic strategies and topical treatments, including lubricating eye drops, topical cyclosporine A, mucin secretagogues, anti-inflammatory agents, eyelid hygiene and punctal occlusion. However, the effectiveness of these treatments can be limited in certain cases, prompting ongoing research into new therapeutic approaches (5).

Leptospermum spp (manuka) honey is a monofloral honey derived from the manuka tree (Leptospermum scoparium), native to New Zealand and eastern Australia. Known for its antimicrobial, antioxidant, and anti-inflammatory properties, it has long been used as a wound healing remedy. Manuka honey inhibits the propagation of inflammatory cells and promotes the proliferation of fibroblasts and epithelial cells, which are essential for tissue repair and regeneration (6, 7). Also, compared with other types of honey, manuka honey has a higher content of polyphenolic compounds, making its anti-inflammatory and antioxidant properties stronger (8, 9).

In ophthalmology, two different concentrations of manuka honey have been approved for treating dry eye disease and meibomian gland dysfunction (MGD): Optimel Manuka+ Dry Eye Drop (Leptospermum spp. honey, 165 mg/g, Melcare Biomedical Pty Ltd, Mt Cotton, Australia) and Optimel Manuka+ Forte Eye Gel (Leptospermum spp. honey, 980 mg/g, Melcare Biomedical Pty Ltd, Mt Cotton, Australia) (10). Additionally, there are studies indicating the possible use of manuka honey for treating persistent corneal epithelial defects and corneal edema following surgery (11, 12).

Since cataract surgery is one of the most common procedures in ophthalmology and dry eye is a frequent postoperative complication, this study aims to evaluate the effect of manuka honey eye drops on postoperative dry eye disease following cataract surgery, in comparison to artificial tears containing sodium hyaluronate.

## Methods

A prospective non-randomized controlled clinical study was conducted at a public tertiary hospital in Madrid, Spain, from March 2023 to June 2024. Participants were consecutively enrolled during routine cataract surgery appointments and were assigned to either the Manuka honey group or the sodium hyaluronate group based on a consecutive allocation scheme. The first set of enrolled patients received Manuka eye drops, and subsequent patients were assigned to the control group.

Inclusion criteria were age of at least 18 years old, cataract requiring surgery and acceptance of voluntary participation as well as signing of informed consent. Exclusion criteria included: presence of any ocular surface or conjunctival pathology, use of other topical treatments (such as antiglaucomatous eyedrops) and previous eye surgery on the same eye.

The variables collected and analyzed in this study included demographic data such as age, sex, eye laterality, and best-corrected visual acuity (BCVA) in logMAR scale. Subjective symptoms were assessed through the Ocular Surface Disease Index (OSDI) and CAT-QUEST 9SF questionnaires. Objective parameters were measured using the Pentacam^®^ and Keratograph^®^ 5M (K5M) (OCULUS, Wetzlar, Germany). With the Pentacam^®^, we evaluated corneal parameters including K1, K2, KMAX, astigmatism axis and power, as well as central corneal thickness. Non-invasive tear break-up time (NIBUT) and conjunctival redness were assessed using the Keratograph^®^ 5M.

Evaluations were carried out over four visits: baseline preoperatively, 1 day after surgery, 1 week (7–10 days) after surgery and 1 month (4–6 weeks) after surgery.

Participants were assigned to either the Manuka honey group or the sodium hyaluronate group (control) based on a consecutive allocation scheme: the first 30 eyes received Optimel Manuka+ Dry Eye Drop^®^ (Leptospermum spp. honey, 165 mg/g), while the remaining 30 eyes were treated with Eyestil SYNFO^®^ (Sodium hyaluronate 0.2%, xanthan gum 0.2%, glycine, and betaine).

The treatment regimen in this study involved the administration of one drop of either Manuka honey or sodium hyaluronate, in addition to standard postoperative care, four times daily (approximately every six hours), beginning on the day of cataract surgery and continuing until the 1-month follow-up visit. To ensure consistency, written instructions for the application of the drops were provided to all patients. Standard postoperative care included the use of tobramycin and dexamethasone eye drops every three hours during the first week, followed by a tapered regimen of dexamethasone over the subsequent four weeks. Routine follow-up visits were conducted to monitor for complications and ensure proper recovery. All patients included in the study received a monofocal intraocular lens with distance vision as the refractive target.

The primary outcome of the study was the change in Ocular Surface Disease Index (OSDI) score from baseline to 1 month postoperatively. Secondary outcomes included conjunctival redness, non-invasive tear break-up time (NIBUT), and CATQUEST-SF9 questionnaire scores.

Statistical analyses were performed using SPSS version 29.0.2 (IBM, Armonk, New York, USA). Categorical variables are presented as percentages, while continuous variables are expressed as means ± standard deviation. For comparisons of categorical variables, the Chi-square test or, when necessary, Fisher’s exact test was employed.

For continuous quantitative variables, normality was evaluated using the Kolmogorov-Smirnov test. If normality was confirmed, either the Student’s t-test or analysis of variance (ANOVA) was applied. In cases where variables did not meet normality assumptions or were presented as ordinal variables, non-parametric tests, such as the Mann-Whitney U test or the Kruskal-Wallis H test, were used. Adjusted analyses were performed using analysis of covariance (ANCOVA), including baseline values and sex as covariates for OSDI, conjunctival redness, and NIBUT. Repeated-measures analyses were also conducted to assess changes over time and differences in temporal trends between groups, applying Greenhouse–Geisser corrections when appropriate. Effect sizes and 95% confidence intervals were reported for adjusted analyses. A p-value < 0.05 was considered statistically significant.

This study received approval from the hospital’s Ethics Committee, and all procedures were conducted in accordance with ethical guidelines for human research. All participants were verbally informed about the study and provided written informed consent prior to their participation. Additionally, data collection and processing were performed with strict adherence to maintaining participant anonymity. Neither patients nor the public were involved in the design, conduct, or reporting of this research.

## Results

A total of 60 eyes from 60 patients were included in the study. Of these, 2 cases were excluded from the final analysis due to intraoperative or postoperative complications (posterior capsule rupture and positive Seidel test requiring reoperation), and 5 patients were lost to follow-up. Therefore, 53 eyes from 53 patients were included in the final analysis: 25 in the Manuka group and 28 in the control group.

The following variables demonstrated a normal distribution: conjunctival redness, mean non-invasive tear breakup time (NIBUT), Ocular Surface Disease Index (OSDI) scores from the first three visits, keratometry readings (K1, K2, and Kmax), astigmatism, and central corneal thickness. In contrast, the first rupture NIBUT across all four visits and the OSDI score at the 1-month visit did not follow a normal distribution.

Overall, 35 were female and 18 were male, with a significantly different distribution between the two groups. The mean age of the patients who underwent surgery was 71.4 ± 7.6 years. The mean BCVA (logMAR scale) was 0.36 ± 0.17, improving to 0.04 ± 0.06 at the final visit (**Table 1**).

**Table 1:** Descriptive data for the Manuka and control groups, along with the p-value representing the statistical differences between the two groups.

Descriptive data	Manuka (n = 25)	Control (n = 28)	p Difference
Age	71.83 ± 6.48	70.17 ± 7.50	p = 0.394
Sex	Male 3 (12%) Female 22 (88%)	Male 15 (53.6%) Female 13 (46.4%)	p = 0.002
Eye	Right 15 (60%) Left 10 (40%)	Right 14 (50%) Left 14 (50%)	p = 0.465
BCVA Baseline	0.35 ± 0.14	0.36 ± 0.19	p = 0.826
BCVA 1 Month	0.05 ± 0.06	0.04 ± 0.05	p = 0.367
BCVA Difference	0.33 ± 0.21	0.32 ± 0.16	p = 0.945

BCVA, Best Corrected Visual Acuity (logMAR Scale).

**Figure 1:**
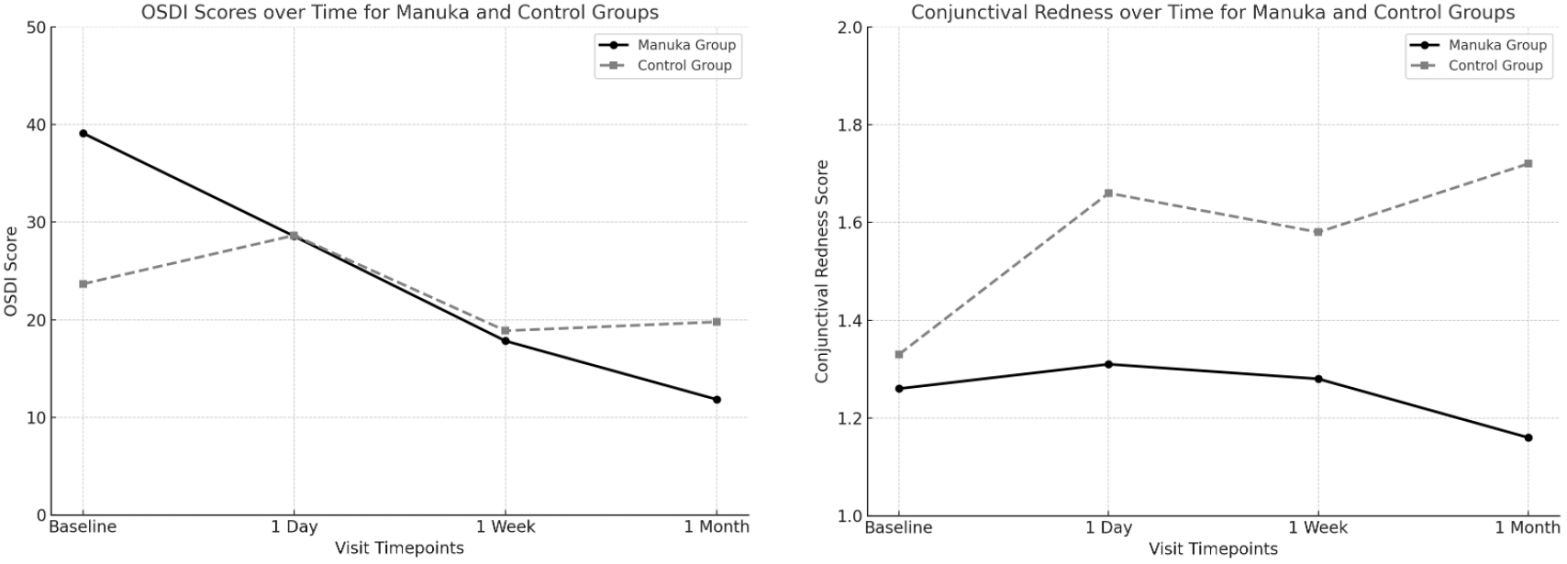
OSDI scores and conjunctival redness over time comparing the Manuka and control groups. Left: OSDI scores. At baseline, the Manuka group exhibited significantly higher OSDI scores compared to the control group (p = 0.005). However, during the month following surgery, the Manuka group demonstrated a greater subjective improvement, reflected in a lower OSDI score at 1 month post-surgery compared to the control group (p = 0.044). Right: Conjunctival redness. At baseline, no statistically significant differences were observed between the groups. However, throughout the postoperative period, the Manuka group showed a reduction in conjunctival redness, while the control group exhibited an increase. The maximum difference between the groups was observed at 1 month post-surgery (p < 0.001).

Concerning the OSDI questionnaire score, Manuka group showed a significantly higher baseline OSDI compared to the control group (39.1 ± 22.6 vs 23.7 ± 14.1; p = 0.005). However, at 1 month postoperatively, OSDI scores were significantly lower in the Manuka group than in the control group (11.8 ± 10.8 vs 19.8 ± 17.8; p = 0.044). Overall, there was a mean improvement of -15.3 ± 22.0 one month after surgery. This improvement was -27.3 ± 20.3 in the Manuka group (p < 0.001) and -4.3 ± 17.4 in the control group (p = 0.214), with a significant difference between the two groups (p < 0.001) (**Figure 1**; **Table 2**).

**Table 2:** Comparison of OSDI scores between the two groups across four follow-up visits.

OSDI	Baseline	1 day	1 week	1 month	Difference (1 month - baseline)	P (Baseline – 1 month)
Manuka	39.11 ± 22.62	28.55 ± 22.23	17.82 ± 15.65	11.83 ± 10.79	- 27.28 ± 20.28	p < 0.001
Control	23.66 ± 14.10	28.64 ± 21.97	18.87 ± 16.13	19.77 ± 17.75	- 4.27 ± 17.41	P = 0.214
Total	31.90 ± 20.81	28.60 ± 21.86	18.37 ± 15.75	15.95 ± 15.21	-15.33 ± 21.97	p < 0.001
p Difference	p = 0.005	p = 0.988	p = 0.822	p = 0.044*	p < 0.001	

The p-values refer to the significance levels from the Student’s t-test, while the variable marked with an asterisk (*) was analyzed using the Mann-Whitney U test.

P-values at individual time points are presented for descriptive purposes only.

After adjusting for baseline OSDI and sex, the Manuka group showed significantly lower OSDI scores at 1 month compared to the control group (adjusted mean difference: -18.74; 95% CI: -28.77 to -8.71; p = 0.007). Baseline OSDI was significantly associated with OSDI at 1 month (p = 0.002), whereas sex was not (p = 0.143). A repeated-measures analysis demonstrated a significant interaction between time and treatment group (Greenhouse–Geisser corrected, p = 0.003), indicating that the evolution of OSDI scores over time differed between groups. A significant effect of time was also observed (p < 0.001), reflecting overall postoperative improvement.

The CATQUEST-SF9 questionnaire demonstrated a statistically significant overall improvement across all nine items at one month post-surgery. For eight of the items, the p-value was < 0.001. However, item C.1, which evaluated difficulties related to reading, exhibited a relatively smaller improvement, with only 45.5% of patients reporting progress. The mean score for this item decreased from 2.2 ± 1.0 to 1.6 ± 0.73 (p = 0.004).

No statistically significant differences were observed between the groups at one month post-surgery for any of the questionnaire items. In terms of difficulties with vision in daily activities, 87.5% of patients in the Manuka group and 78.6% of patients in the control group showed improvement (p = 0.385). Likewise, in terms of overall satisfaction with current vision, 100% of patients in the Manuka group and 92.9% of patients in the control group reported improvement (p = 0.273).

In the pre- and post-surgery analysis, item C.3, which evaluated distance vision, demonstrated a significantly greater improvement in the Manuka group (-1.2 ± 0.8) compared to the control group (-0.6 ± 0.9) (p = 0.029). Similarly, item C.6, which assessed near vision, showed a greater improvement in the Manuka group (-1.43 ± 1.0) compared to the control group (-0.7 ± 0.8) (p = 0.016). Both variables exhibited significant baseline differences, with higher initial scores observed in the Manuka group.

In terms of objective conjunctival redness measured with K5M, the baseline mean was 1.3 ± 0.3, with a worsening of 0.2 ± 0.5 one month after surgery. In the Manuka group, redness decreased by -0.1 ± 0.3, while it increased by 0.4 ± 0.6 in the control group (p < 0.001). After adjusting for baseline conjunctival redness and sex, the Manuka group showed significantly lower conjunctival redness at 1 month compared to the control group (adjusted mean difference: -0.56; 95% CI: -0.91 to -0.21; p = 0.014). Baseline redness was significantly associated with postoperative redness (p < 0.001), whereas sex was not (p = 0.859). Repeated-measures analysis did not show a significant interaction between time and treatment group (Greenhouse–Geisser corrected, p = 0.065) (**Figure 1**; **Table 3**).

**Table 3:** Assessment of conjunctival redness in the Manuka and control groups.

Conjunctival redness	Baseline	1 day	1 week	1 month	Difference (1 month - baseline)	P (Baseline – 1 month)
Manuka	1.26 ± 0.30	1.31 ± 0.49	1.28 ± 0.42	1.16 ± 0.35	- 0.09 ± 0.31	p = 0.165
Control	1.33 ± 0.33	1.66 ± 0.64	1.58 ± 0.61	1.72 ± 0.66	0.39 ± 0.55	p < 0.001
Total	1.30 ± 0.32	1.49 ± 0.60	1.44 ± 0.55	1.48 ± 0.61	0.18 ± 0.52	p < 0.001
p Difference	p = 0. 394	p = 0.029	p = 0.052	p < 0.001	p < 0.001	

P-values at individual time points are presented for descriptive purposes only.

NIBUT did not demonstrate statistically significant differences in either the time to first break or the mean break time between the two groups at any of the four postoperative visits. In the comparison between baseline and one month post-surgery, the Manuka group exhibited an improvement (increase) in the time to first tear break of 1.8 ± 8.7 seconds, whereas the control group showed a decrease of -0.9 ± 9.0 seconds (p = 0.143). Similarly, the mean break time increased by 1.2 ± 7.5 seconds in the Manuka group, while a decrease of -1.5 ± 6.7 seconds was observed in the control group (p = 0.091). After adjusting for baseline values and sex, no statistically significant differences were observed between groups in NIBUT at 1 month, either for the first measurement (adjusted mean difference: 0.97; 95% CI: -3.61 to 5.56; p = 0.404) or for the mean NIBUT (adjusted mean difference: 2.58; 95% CI: -1.24 to 6.40; p = 0.285). Baseline NIBUT was not significantly associated with postoperative values in either analysis (p = 0.736 and p = 0.894, respectively), and sex was also not a significant predictor. Repeated-measures analysis showed no significant interaction between time and treatment group for NIBUT, either for the first measurement (Greenhouse–Geisser corrected, p = 0.202) or for mean NIBUT (p = 0.571), indicating a similar temporal evolution in both groups. No significant effect of time was observed for either parameter (p = 0.611 and p = 0.500, respectively) (**Table 4**).

**Table 4:** Non-invasive tear break-up time (NIBUT), including both first tear and mean tear time (measured in seconds), compared between the Manuka and control groups.

NIBUT	Baseline	1 day	1 week	1 month	Difference (1 month - baseline)	P (Baseline – 1 month)
NIBUT first (s)						
Manuka	7.75 ± 4.86	7.82 ± 4.37	6.90 ± 4.66	9.67 ± 7.07	1.75 ± 8.72	p = 0.330*
Control	8.72 ± 6.41	7.57 ± 4.88	7.03 ± 5.92	7.79 ± 5.69	-0.93 ± 8.96	p = 0.622*
Total	8.28 ± 5.73	7.69 ± 4.59	6.96 ± 5.28	8.58 ± 6.32	0.20 ± 8.88	p = 0.764*
p Difference	p = 0.793*	p = 0.591*	p = 0.689*	p = 0.287*	p = 0.143	
NIBUT med (s)						
Manuka	13.90 ± 4.21	14.14 ± 3.97	14.62 ± 5.30	14.80 ± 5.49	1.20 ± 7.54	p = 0.231
Control	15.22 ± 5.04	14.78 ± 4.45	12.52 ± 5.64	13.74 ± 5.38	-1.48 ± 6.67	p = 0.117
Total	14.62 ± 4.68	14.47 ± 4.19	13.55 ± 5.52	14.19 ± 5.40	- 0.34 ± 7.11	p = 0.364
p Difference	p = 0.150	p = 0.146	p = 0.093	p = 0.245	p = 0.091	

P-values at individual time points are presented for descriptive purposes only. The p-values refer to the significance levels from the Student’s t-test, while the variable marked with an asterisk (*) was analyzed using the Mann-Whitney U test.

Corneal tomography performed with the Pentacam^®^ revealed a mean increase in total astigmatism of 0.04 ± 0.37 diopters, an increase in Kmax of 0.21 ± 1.23 diopters, and a corneal thickness increase of 3.5 ± 17.2 µm one month post-surgery. None of these changes reached statistical significance, and no significant differences were observed between the groups for any of the parameters analyzed.

A correlation analysis was conducted between BCVA, OSDI, CAT-QUEST, conjunctival redness, NIBUT, and Pentacam^®^ parameters. The analysis revealed that only the OSDI and CAT-QUEST scores were significantly correlated (Spearman’s Rho, p < 0.05), indicating a relationship between subjective measures of visual function and patient-reported outcomes. No statistically significant correlations were found among the other variables.

## Discussion

This prospective controlled study suggests that Manuka honey eye drops are associated with greater improvement in postoperative dry eye symptoms, as measured by the OSDI, and with reduced conjunctival redness compared to sodium hyaluronate. These findings were observed despite baseline differences between groups and remained significant after adjustment for potential confounders, including baseline values and sex.

The baseline OSDI score was significantly higher in the Manuka group compared to the control group, but one month after cataract surgery, there was a reversal, with the Manuka group showing a significantly lower OSDI score than the control group (p < 0.05). A statistically significant improvement (reduction) in OSDI score was also observed only in the Manuka group when compared to baseline at one month post-surgery. These findings, together with the adjusted analyses, suggest that the observed effect cannot be fully explained by baseline differences.

Several studies have examined changes in OSDI scores following cataract surgery. However, these studies show high heterogeneity, and there is no consistent evidence of worsening ocular surface symptoms one month post-surgery (13, 14). It is important to note that while the total OSDI score may not change significantly, OSDI items related to visual function may mask increases in ocular discomfort and environmental trigger-related symptoms (15). In our study, there was no significant correlation between BCVA and OSDI score, but visual function items were not analyzed separately.

The greater subjective improvement in the Manuka group aligns with previous studies on this subject, although evidence remains scarce (8). For instance, Tan J. et al. (16) reported a similar improvement in OSDI scores in dry eye patients, with the Manuka-treated group improving by -19.6 ± 10.9 compared to -10.7 ± 7.1 in the control group (p = 0.005), results that are consistent with our findings in cataract surgery patients (p = 0.001). Manuka honey has also been studied in patients with blepharitis (17), MGD-associated dry eye (10), and contact lens users (18). The improvements in OSDI scores across these various conditions further support our findings of symptomatic relief in cataract surgery patients.

Regarding the CATQUEST-SF9 questionnaire, both groups showed improvement across all nine questions, with no significant differences between them. Question C.1, which refers to difficulties reading newspaper text, showed the least improvement (only 45.5% of subjects improved), likely related to the implantation of monofocal intraocular lenses (19). Although items C3 and C6 of the CATQUEST-SF9 questionnaire showed greater improvement in the Manuka group, these findings should be interpreted with caution. Both items presented significantly higher baseline scores in the Manuka group, suggesting a potential regression to the mean effect. Importantly, no significant differences between groups were observed at one month postoperatively. Therefore, these results are unlikely to reflect a true treatment effect and should not be attributed to the efficacy of Manuka eye drops.

As for objective conjunctival redness, significant differences were observed in favor of the Manuka group one month post-surgery. Patients in this group exhibited redness levels similar to baseline, while the control group showed a significant increase. This may be due to the anti-inflammatory effects of Manuka honey, which can reduce cytokines such as IL-1ß, IL-6, and TNF-α—molecules typically elevated in inflammatory ocular surface conditions like dry eye disease (20). All patients received postoperative corticosteroids, which may have influenced ocular surface inflammation; however, their uniform use across groups makes it unlikely that they explain the observed differences, although a residual confounding effect cannot be excluded.

Several methods have been described to measure redness associated with dry eye disease. Recent advances in these methods have focused on photographic image processing through various techniques, including the K5M, which appears to show good sensitivity to vasoconstrictor-induced changes, although it is less sensitive to dry eye disease-related changes than the validated redness scale (VBR) (21). In this study, considering the variability of this technique, we conducted a before-and-after comparison for each patient, with all tests performed by the same two specialists to reduce inter-observer variability.

In the assessment of NIBUT, no statistically significant differences were observed between the groups studied. However, the Manuka group exhibited an improvement, evidenced by an increase in both the first tear break-up time (p=0.143) and the mean tear break-up time (p=0.091), compared to the control group. The NIBUT measurements obtained with the K5M demonstrated good repeatability and reproducibility in patients without dry eye disease diagnosis, such as those included in this study (22). Typically, NIBUT does not display significant changes one month following cataract surgery (23).

When compared to the existing literature on Manuka, it is noteworthy that studies conducted at 15 and 28 days (16, 18) reported no significant improvements in NIBUT, while assessments at 8 weeks and 90 days (10, 17) revealed statistically significant enhancements. Future investigations should aim to determine whether Manuka-treated patients experience improvements in tear film stability at 2 to 3 months post-cataract surgery. Additionally, conducting a larger sample size analysis at the 1-month mark could help confirm the statistical significance of these observed differences.

The final variables analyzed pertained to corneal morphology, as measured by tomography. No statistically significant differences were identified either between the study groups or when comparing the baseline measurements with those obtained one month postoperatively. Surgically induced astigmatism (SIA) is a well-documented consequence of cataract surgery, primarily attributable to alterations in the posterior corneal curvature (24). These corneal changes are not uniform among surgeons and are predominantly influenced by the angulation of the primary surgical incision (25). In this study, the procedures were performed by a large number of surgeons operating under diverse baseline conditions, which likely accounts for the absence of significant pre- and post-surgical differences in corneal morphology.

This study has some limitations. The relatively small sample size and short follow-up may have reduced statistical power and limited the assessment of long-term outcomes. In particular, previous studies on Manuka honey have reported significant improvements after longer treatment durations (8–12 weeks), whereas shorter follow-up periods (15–28 days) often show no significant differences. Therefore, the one-month follow-up in this study may have been insufficient to detect changes in certain parameters, such as tear film stability. In addition, patients were allocated using a consecutive, non-randomized scheme, which may have introduced selection bias and contributed to baseline imbalances between groups, potentially affecting the comparability of the results. Variability in surgical techniques and reliance on patient-reported questionnaires could also have influenced the results, although objective measures were included to balance this. In addition, the study was not prospectively registered as clinical trial, reflecting its design as a low-risk, non-randomized controlled investigation.

Despite these limitations, the study has notable strengths. It is among the first prospective controlled investigations to examine Manuka honey eye drops after cataract surgery, combining validated subjective questionnaires with objective ocular surface assessments. The use of a real-world surgical setting with consecutive patient allocation enhances the clinical applicability of the findings, which consistently support the potential value of Manuka honey as an adjunctive treatment for postoperative dry eye disease.

In conclusion, Manuka eye drops were associated with greater improvement in dry eye symptoms and conjunctival redness following cataract surgery compared to sodium hyaluronate. These findings suggest that using Manuka eye drops may represent a promising adjunctive option for the management of postoperative dry eye. However, given the non-randomized design and baseline imbalances, these results should be interpreted with caution and considered hypothesis-generating.

## Data Availability

The original contributions presented in the study are included in the article/supplementary material. Further inquiries can be directed to the corresponding authors.
